# Yoga and schizophrenia—a comprehensive assessment of neuroplasticity: Protocol for a single blind randomized controlled study of yoga in schizophrenia

**DOI:** 10.1097/MD.0000000000017399

**Published:** 2019-10-25

**Authors:** Shivarama Varambally, Ganesan Venkatasubramanian, Ramajayam Govindaraj, Venkataram Shivakumar, Thrinath Mullapudi, Rita Christopher, Monojit Debnath, Mariamma Philip, Rose Dawn Bharath, BN Gangadhar

**Affiliations:** a Department of Psychiatry; b Department of Human Genetics; c Department of Neurochemistry; d Department of Biostatistics; e Department of Neuroimaging and Interventional Radiology, National Institute of Mental Health and Neurosciences, Bangalore, India.

**Keywords:** schizophrenia and yoga, yoga and brain derived neurotrophic factor, yoga and emotion processing, yoga and plasma oxytocin, yoga and psychopathology

## Abstract

**Introduction:**

Schizophrenia is one of the most severe mental disorders with a prevalence of about 1% and a leading cause of disability among young adults. Pharmacotherapy is the mainstay in the management of schizophrenia. However, even with the best of medication, several problems like refractoriness, negative symptoms, frequent relapses, and cognitive impairments persist.

**Methods:**

This is a randomized-controlled clinical study including patients from an urban tertiary hospital and a semi-urban community center, with a between-group, repeated-measures, longitudinal design. This study will recruit 160 patients with DSM 5 diagnosis of schizophrenia who are on stable medication for a minimum of 6 weeks; they will be randomly assigned into 2 arms viz., yoga therapy (YT), and treatment-as-usual (TAU) with 80 patients in each arm. Participants will undergo Clinical, Laboratory, and Radiological assessments at baseline and at intervals of 1 month, 3 months, and 6 months from the baseline. It is hypothesized that yoga will improve psychopathology and emotion processing, increase serum brain derived neurotrophic factor (BDNF) and plasma oxytocin levels and effect changes in cerebral activation in areas of the brain associated with schizophrenia.

**Discussion:**

This study aims to measure the efficacy of a Yoga-based intervention as an adjunct in patients with schizophrenia as well as the mechanisms of these effects.

**Trial registration:** Registered retrospectively with Clinical Trial Registry – India (CTRI) with registration number CTRI/2017/08/009219.

## 1 Introduction

Schizophrenia is a severe mental illness with a tendency to have a chronic course. Antipsychotic medication is the standard treatment, but has its own limitations, especially with negative and cognitive symptoms showing little or no response to antipsychotics. Also, while the classical antipsychotics had limitations of disabling side effects like extra pyramidal side effects (EPS) and tardive dyskinesia, the newer generation drugs have varied metabolic side effects which may in turn worsen the overall well-being. Also, currently there is a significant drug deadlock in schizophrenia.^[1]^ Hence there is a continuous quest for additional modalities of treatment which can alleviate the impairment caused by the illness.

Yoga which is a part of the ancient heritage of India has been shown to have significant positive effects in stress, cognitive functioning, and neuropsychiatric disorders such as anxiety and depression.^[2–4]^ Yoga and pranayama also have demonstrated efficacy in reducing metabolic parameters such as cortisol in stress-related disorders, serum insulin, and lipid profile in metabolic disorders.^[5,6]^

There is also recent evidence showing that yoga facilitates neuroplasticity in disorders such as depression^[7]^ and that yoga can increase neurotransmitter levels in certain brain regions.^[8,9]^ Preliminary studies also show that yoga and meditative practices may increase cortical thickness,^[10]^ and increase volume of grey matter in parts of the brain important for memory and cognition.^[11,12]^ Thus, yoga based interventions have the potential to ameliorate the residual symptoms in schizophrenia and adaptively modulate the neuroplasticity abnormalities in this disorder.

### 1.1 Yoga in schizophrenia

Yoga-based intervention has been shown to significantly improve clinical symptoms and functioning in patients with schizophrenia.^[13,14]^ We have developed and validated a specific module of yoga for schizophrenia^[15]^ and demonstrated significant improvement in symptoms (particularly negative symptoms and emotion recognition deficits) as well as real-life functioning.^[16–18]^ It is noteworthy that some of this clinical work has been recognized by the expert panel of National Institute of Health and Care Excellence (NICE) (UK) in the 2014 guidelines for Schizophrenia as “high quality” evidence for use of yoga in schizophrenia, leading to a recommendation for the use of yoga as a complementary treatment in schizophrenia for the first time.

In a study on stable patients with schizophrenia, it was found that serum oxytocin levels rose after the practice of yoga along with improvement in clinical symptoms.^[19]^ However, this is preliminary, and more research needs to be done to systematically elucidate the effects of yoga in schizophrenia, the biological mechanisms involved, and the role of neuroplasticity. The recent NICE guidelines also called for more systematic research into the effects of physical therapies such as yoga in psychosis.

To summarize, there are many promising leads that support the utility of yoga-based interventions as an important add-on option for treating schizophrenia patients; a comprehensive evaluation of clinical benefits with concurrent evaluation of its potential neurobiological correlates will further strengthen the translational implications of yoga in schizophrenia. The proposed study is an attempt to fill the knowledge gap in this area. Our previous studies (including waitlist and physical exercise arms) have shown the efficacy of add-on yoga therapy for patients with schizophrenia. The current study is aimed at replicating the clinical results as well as examining the biological mechanisms involved.

### 1.2 Objectives

#### 1.2.1 Primary objectives

1. To systematically assess the effect of yoga-based intervention as an add-on treatment in schizophrenia on psychopathology and social cognition using a randomized controlled design.
2. To investigate the neurobiological mechanisms of action of yoga in schizophrenia by assessing serum brain derived neurotrophic factor (BDNF), plasma oxytocin and functional magnetic resonance imaging (fMRI).

#### 1.2.2 Secondary objectives

1. To assess the effect of yoga-based intervention as an add-on treatment in schizophrenia on quality of life, perceived stress levels, and socio-occupational functioning.
2. To investigate the effects of yoga-based intervention on serum cortisol, serum insulin like growth factor-1 (IGF-1), expression of BDNF and oxytocin gene in peripheral lymphocytes in patients with schizophrenia.

### 1.3 Hypotheses

#### 1.3.1 Primary hypotheses

In patients with schizophrenia, add-on yoga-based intervention will lead to:

1. Improvement in psychopathology and emotion processing in patients with schizophrenia.
2. Increase in serum BDNF and plasma oxytocin levels in patients with schizophrenia.
3. Changes in cerebral activation in areas of the brain associated with symptoms of schizophrenia (limbic area and prefrontal cortex).

#### 1.3.2 Secondary hypotheses

In patients with schizophrenia, add-on yoga-based intervention will lead to:

1. Improvement in quality of life, perceived stress levels, and socio-occupational functioning in patients with schizophrenia.
2. Decrease in serum cortisol, increase in serum IGF-1, and significantly enhanced expression of BDNF and oxytocin gene in peripheral lymphocytes.

## 2 Materials and methods

The initial study protocol proposed to randomize patients with schizophrenia on stabilized medication into 3 arms—one with yoga as an add-on intervention, a second arm with a module of physical exercise as an add-on intervention, and a waitlist arm with no additional intervention. However, a pilot phase found that there were significant difficulties in patient acceptance for the exercise arm leading to refusal of randomization in several subjects. Therefore, the protocol was modified in consultation with the funding agency and the Trial Advisory Committee to a two-arm study with the physical exercise arm being dropped. In addition, a community recruitment center was added (District Mental Health Services, Ramanagara, Karnataka, India). Institutional Ethics Committee approval was then obtained for the revised study. The above modification has been communicated to the Trial Registry where the study is registered (Clinical Trial Registry—India).

The source of patients is the outpatient and inpatient services run by the Department of Psychiatry, National Institute of Mental Health and Neurosciences (NIMHANS), Bengaluru, India and the District Mental Health Services, Ramanagara, Karnataka.

### 2.1 Selection of subjects

#### 2.1.1 Inclusion criteria

(1) Diagnosis of schizophrenia (DSM—V).
(2) Clinical Global Impression–Severity^[20]^ (CGI-S) Score ≥3.
(3) Age range: 18 to 45 years.
(4) Both sexes.
(5) Written informed consent.

#### 2.1.2 Exclusion criteria

(1) Risk of harm to self (e.g., suicidal risk) or others (e.g., aggression).
(2) Need for electroconvulsive therapy.
(3) Subjects who are already practicing/recent practice of yoga/meditation.
(4) Comorbid substance dependence in the past 6 months or substance abuse in the past 1 month as per DSM-V, except nicotine.
(5) Significant neurological disorder including seizure disorder or recent head injury.
(6) Family history of neurological disorder that may complicate diagnosis (for example Huntington chorea).
(7) Pregnancy or postpartum (<6 weeks after delivery).

### 2.2 Clinical assessment

The demographic and clinical information is collected using structured scales and proforma. Diagnosis of schizophrenia is made as per Diagnostic and Statistical Manual of Mental Disorders^[21]^ (DSM-5) criteria and Mini-International Neuro psychiatric Interview Plus^[22]^ (M.I.N.I.). The clinical diagnosis is confirmed independently by a qualified psychiatrist. Psychopathology is assessed using the Scale for Assessment of Positive Symptoms^[23]^ (SAPS); Scale for Assessment of Negative Symptoms (SANS),^[23]^ and Brief Psychiatric Rating Scale (BPRS).^[24]^ Social cognition is assessed by the Tool for Recognition of Emotions in Neuropsychiatric disorders^[25]^ (TRENDS). Social and occupational performance is rated on the Social Occupational Functioning Scale^[26]^ (SOFS) and Quality of life on the World Health Organization's quality of life assessment^[27]^ (WHOQUOL-BREF). Perceived stress is rated using the Cohen Perceived Stress Scale.^[28]^ Adverse effects of antipsychotics are assessed using the Simpson Angus Scale for Extrapyramidal Symptoms^[29]^ (SAS) and the Abnormal Involuntary Movements Scale (AIMS).^[30]^

#### 2.2.1 Sample size estimation

For evaluating the primary objectives, the optimal sample size was estimated using the principles and methods^[31]^ for the assessment of primary parameters (SAPS, SANS, and BPRS scores, serum BDNF, Oxytocin, TRENDS fMRI). Based on approximation and estimation from the previous data on the clinical effects of add-on yoga therapy on these parameters in schizophrenia patients^[7,16,18]^ supporting a medium effect size for yoga-based intervention, it was estimated that, for an allocation ratio of 1, a sample size of at least 67 schizophrenia patients in each arm for the pre- versus post- comparisons of patients will be required to detect a two-tailed significant difference of *α* = 0.01 with an estimated 90% power. The study plans to recruit 80 patients in each arm (a total of 160 patients) to account for potential drop-outs.

The subset sample size of 50 in each arm for imaging studies (in each group—pre as well post brain scans) is sufficient to perform random effect analysis for fMRI BOLD changes with multiple comparison correction using random field theory based family-wise error correction to control for multiple comparison (http://www.fil.ion.ucl.ac.uk/spm/).

### 2.3 Study design description

The study has received approval from the NIMHANS Human Ethics Committee (No.NIMHANS/DO/104^th^IEC/2016). Patients with schizophrenia attending the outpatient/inpatient services of NIMHANS and the District Mental Health Services, Ramanagara who are stabilized on antipsychotics for at least 6 weeks are approached. Patients fulfilling the selection criteria are requested to give written informed consent by the Post-Doctoral Fellow working in the study. The course of the study, its merits, possible demerits, rights to withdraw from the study at any time are clearly explained in the patient's own language and a copy of the Informed Consent form explaining the same is given to the patient or parent/guardian. Information obtained from subjects will be kept confidential. Consecutive consenting patients at either center are randomly assigned to any one of the 2 groups (1:1 allocation ratio)—yoga group or waitlist group, using computer-generated random numbers and allocation concealment. One of the co-investigators who is a biostatistician (MP) and not involved in assessing subjects generates the random number sequence with a computer and allots the subjects to either group with a serially numbered opaque sealed envelope. Raters/lab personnel are blinded to allocation. As double-blinding is not feasible in yoga studies, the rater/lab personnel being blind to group allocation is the best practice in such studies.^[32]^ All subjects will continue their antipsychotic medications as per their treating psychiatrist till the end of the study. Other than for clinical emergencies, medications are changed for these patients. If change in treatment is necessitated, patients are dropped from the study.

**Figure 1:**
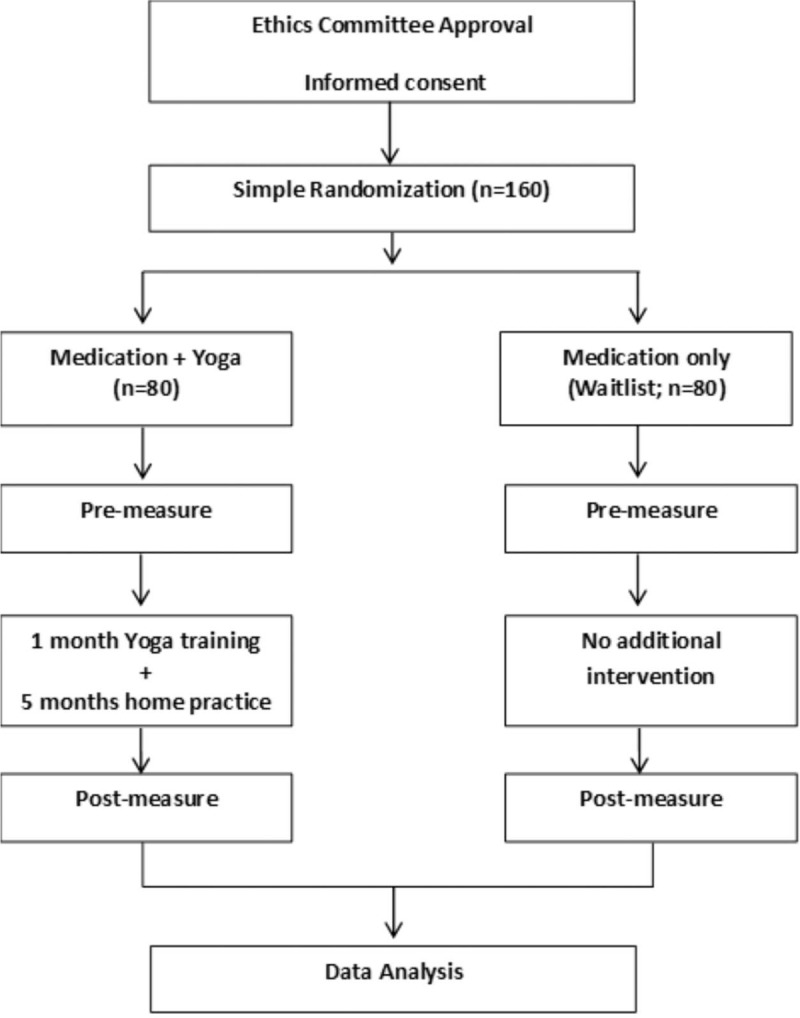
Study outline.

The subjects in the yoga group are trained in a 60 minutes validated yoga module^[15]^ for 1 month (at least 20 sessions) by a trained instructor at the NIMHANS Integrated Centre for Yoga and the community center. These patients are asked to continue the respective practices at home for 5 months at least 3 days a week. Patients are followed up monthly with a booster session, and are provided with treatment manual (including log book), practice video, and monitored by phone. The patients in the waitlist group are asked to continue with the prescribed medications, and requested to be available for assessments. They are offered yoga/exercise as per their choice after completing the 6-month period. The study outline is given in Fig. 1.

### 2.4 Plans for data storage, handling, and statistical analysis

Data storage and handling is under the supervision of a collaborator (biostatistician) from the Department of Biostatistics at NIMHANS. The data is coded and stored under password protection. Data analysis will also be carried out under the supervision of the Principal Investigator and the biostatistician. The person carrying out the analysis will be blind to subjects’ group allocation status. Intention to treat analysis and per protocol analysis would be done to account for the missing data. Interim analysis is planned at the end of 3 years of the study. There is no formal Data Monitoring Committee for the trial. However, a Trial Steering Committee is in place to monitor the conduct of the study. In addition, the Wellcome Trust - DBT India Alliance Expert Review committee reviews the trial annually and suggests modifications if needed. The NIMHANS Human Ethics Committee monitors ethical issues related to the trial.

Primary outcome measures: change in SAPS, SANS, BPRS scores and sub-scores; change in TRENDS accuracy scores (TRACS); change in serum BDNF; change in plasma oxytocin; and differences in BOLD response among study groups, and treatment effects (i.e., baseline vs follow-up comparison).

Secondary outcome measures: change in WHOQUOL-BREF scores; change in Perceived Stress Scale (PSS) scores; change in SOFS scores; change in serum cortisol; change in serum IGF-1; and change in gene expression of BDNF and oxytocin genes.

Physical activity levels of all subjects is obtained using a standard questionnaire and controlled for during analysis. Antipsychotic medication dosage would be recorded and analyzed as a covariate.

Data analysis will be done using SPSS Version 24 (IBM SPSS Statistics for Windows, Version 24.0. Armonk, NY: IBM Corp). Basic details of patients who opt out of the study are recorded, and measures to deal with dropouts, missing data, and non-adherence will be taken in collaboration with the biostatistician. Intent-to-Treat analysis will be done including data of all randomized subjects. Mixed model multivariate analysis will be used. Details of imaging analysis are given in the section on imaging below.

### 2.5 Interventions

For yoga group—Validated Yoga Module for Schizophrenia—duration approximately 60 minutes.^[15]^

Treatment-as-usual (waitlist) group: This group will not receive any additional interventions. They are asked to continue with the prescribed medications, and requested to be available for assessments.

Previous studies in this area have noted changes after 1 month of yoga training and at 3 to 4 months follow up.^[18]^ However, there is no literature on the sustainability of the changes beyond 4 months and reviews on the subject have called for confirmation of the findings and longer follow-up. In this light, the proposed study aims to replicate the findings of the previous studies and extend the follow-up to 6 months. Other than the clinical rating scales, the other assessments are not expected to have significant issue with repeated measurements. Some of the measures which are not expected to change within a short period (SOFS, WHOQUOL-BREF) are to be repeated at baseline, 3 and 6 months only.

#### 2.5.1 Description of some relevant assessments

(1) Tool for Recognition of Emotions in Neuropsychiatric disorders^[25]^ (TRENDS): This is a tool validated for use in the Indian population, which captures the full range and nature of emotional expressions akin to real life situations and can be utilized for behavioral and functional imaging studies in Indian patients. It takes into account variations of age and sex on emotional expressions and is a culture-sensitive tool. This is a culturally sensitive, ecologically valid tool, consisting of 52 static (still) and 28 dynamic (video clip) images (i.e., totally 80 images) of 6 basic emotions—happy, sad, fear, anger, surprise, disgust, and a neutral expression emoted by 4 experienced actors (1 young man, 1 young woman, 1 older man, and 1 older woman).
(2) Socio Occupational Functioning Scale^[26]^ (SOFS): This is a comprehensive, easy to administer measure of social functioning in schizophrenia patients developed in India. The scale has 14 items rated on a 5-point spectrum (1 = no impairment, 5 = extreme impairment). The scale has a 3 factor structure comprising of adaptive living skills, social appropriateness, and interpersonal skills.
(3) Perceived Stress Scale^[28]^ (PSS): The Perceived Stress Scale (PSS) designed by Sheldon Cohen is one of the most widely used psychological instruments for measuring the perception of stress. It is a measure of the degree to which situations in one's life are appraised as stressful. Items are designed to tap how unpredictable, uncontrollable, and overloaded respondents find their lives. The scale also includes a number of direct queries about current levels of experienced stress. The items are easy to understand, and the response alternatives are simple to grasp. Moreover, the questions are of a general nature and hence are relatively free of content specific to any subpopulation group. The questions in the PSS ask about feelings and thoughts during the last month. In each case, respondents are asked how often they felt a certain way.
(4) Serum Cortisol, IGF-1, and BDNF estimation: Venous blood (around 5 mL) is collected from patients in anticoagulant-free tubes around 9 am and serum is separated after 30 minutes by centrifugation (2900 rpm for 15 minutes). Coded serum sample is stored at –80 °C deep freezer. Serum Cortisol, IGF-1, and BDNF levels are measured using Enzyme-linked Immuno Sorbent Assay (ELISA) by commercial reagents according to the manufacturer's instructions.
(5) Plasma Oxytocin estimation: Blood sample is collected around 9 am after overnight fasting. Five cubic centimeter of blood is drawn and collected into chilled EDTA (1 mL/mL of blood) tubes containing aprotinin (500 KIU/mL). Sample is centrifuged at 1600 × *g* for 15 minutes at 2 to 8 °C. Plasma sample is immediately frozen and kept at –80 °C deep freezer till analysis. Plasma oxytocin levels will be measured by ELISA using commercial kits.

Note: All aseptic precautions will be taken while collecting blood to ensure patient safety.

(1) Gene expression studies:
  - Lymphocytes Preparation: Peripheral blood (5 mL) is drawn from the cubital vein into using BD vacutainer. EDTA-treated blood is layered on the Ficoll Paque solution and centrifuged for a short period of time. The lymphocytes are then recovered from the interface between the plasma and the Ficoll-Paque and subjected to short washing steps with a balanced salt solution to remove any platelets, Ficoll-Paque and plasma. The resulting lymphocyte pellet is subjected to RNA preparation procedures. The choice of lymphocytes for gene expression studies is based on support for the usage of lymphocytes as a neural probe with promising utility for studying psychiatric disorders.^[33]^ Moreover, studies have demonstrated that altered T lymphocyte function in schizophrenia as a cellular model to investigate molecular disease mechanisms of this disorder.^[34]^
  - RNA Isolation and Analysis: Total RNA is isolated from lymphocytes using commercial silica membrane based spin column method. In this procedure, the leukocytes are lysed using highly denaturing conditions that immediately inactivate RNases, allowing the isolation of intact RNA. RNA will get bound to the silica membrane during centrifugation, which is then washed and eluted in RNase-free water. RNA concentration is measured photometrically and purity checked through the ratio of optical density at 260 and 280 nm. The quality of the isolated RNA is checked by agarose gel electrophoresis (1% agarose, formaldehyde containing) through interpretation of 18 S and 28 S bands. Only high quality RNA is used for further analysis.
  - cDNA-synthesis and Gene Expression Analysis: Total RNA is reverse-transcribed into first-strand cDNA by using commercial reverse transcriptase with RNase H activity and random hexamer/oligo-dT primers. A ribonuclease activity of RNase H of Reverse Transcriptase specifically degrades only the RNA in RNA: DNA hybrids. cDNA product is used as template for Quantitative real-time Polymerase Chain Reaction. Oxytocin and BDNF gene expression is analyzed quantitatively using TaqMan Gene expression assays with Real-Time PCR system (Applied Biosystems, CA, USA). The assay is validated using relevant endogenous control like GAPDH or β-actin used to normalize for differences in sample RNA added to a reaction.
(2) Magnetic resonance imaging methods

MRI Scanner and related hardware: MRI data is acquired using 3T scanner (Ingenia CX, Philips Healthcare, Best, Netherlands, R5.3.1.2). For task-based functional MRI (fMRI), the following hardware are used: MR compatible display monitor (LCD, 32-in.), SyncBox (Nordic Neuro Lab Inc., Norway) and button response device (Current Designs Inc., PA).

##### 2.5.1.1 MRI scanning: screening and training pre-scan assessments and training

MRI is done on the same day of the clinical assessment and blood sample collection. Patients are provided with the required information about the scanning procedures. They are trained by the clinician-researcher to familiarize themselves with the task-based fMRI experiments. The patients are comprehensively screened for MR safety by the clinical team, followed by on-site screening by the MRI center staff before the initiation of the scan. A fiducial marker (Capsule Vitamin E) is positioned at the right temporal region of the patients for ascertaining laterality of the MRI data. The following MRI scans are acquired:

Structural MRI (T1): T1-weighted single-shot 3D turbo field echo (TFE) image is acquired. Other parameters of the structural MRI scan are: TR = 2500 ms, TE = 2.9 ms, Flip angle = 9°, FOV = 256 mm covering the whole brain including cerebellum and brain stem, slice thickness = 1 mm without interslice gap, Matrix = 256 × 256; Voxel size = 1  mm × 1 mm × 1 mm, acceleration factor (SENSE) = 2, 192-sagittal slices.

Functional MRI (fMRI): The functional MRI scans (blood oxygenation level-dependent [BOLD] sensitive echo planar imaging [EPI] sequence) comprise of resting-state fMRI and task-based fMRI for emotion processing (emotions–matching and labeling task [E-MALT]). A second-order pencil beam shimming covering the entire brain is applied to facilitate homogenization of signal acquisition to minimize signal loss in certain susceptible regions like orbitofrontal cortex. Field map and phase-encoding EPI reference scans (with opposing polarity) are acquired along with other functional MRI scans (i.e., resting/E-MALT) for correction of geometrical distortion (if any) during image processing.

Resting-state fMRI (RS-fMRI): RS-fMRI is acquired in the eyes closed condition, and the participants would be instructed to relax, not think anything actively, and lay still. The scan is acquired with following parameters: TR = 2200 ms; TE = 28 ms; flip angle = 80°; slice-thickness = 3 mm; slice-order: ascending; slice-number = 44; Gap = 0.3 mm; Matrix = 64 × 64 × 64 mm^3^, FOV = 211 × 211, voxel = 3.0 mm isotropic with 275 dynamic scans.

##### 2.5.1.2 Task fMRI-1: EMALT paradigm

The experimental paradigm consists of human faces depicting fear, anger, sad, and disgust emotions obtained from a validated tool culturally appropriate for Indian subjects—Tool for Recognition of Emotions in Neuropsychiatric Disorders (TRENDS).^[25]^ To explore the complex emotion processing function the TRENDS experiment was modified to incorporate emotion matching and labeling tasks based on pre-existing studies.^[35]^ In this experiment subjects perform 3 tasks; matching of facial emotions, labeling of facial emotions and a control task. In the matching task subjects are presented with a target face along with 2 other faces on the same screen. They are asked to select which one of 2 faces presented on the same screen expressed the same emotion as that of target face by pressing the buttons of the button response box. In the labeling condition, subjects view the same target face but have to judge which of two linguistic labels, given below the target best describes the target facial emotion. For each affect condition, 32 different images are used, 8 per block, 4 of each sex, and 4 of old age and 4 of young. Each block consists of images of either fear and anger/sadness and disgust of low and high intensity in separate blocks. For control task, 6 different geometric forms are used.

The paradigm consists of a total of 16 experimental blocks, 4 blocks each of matching and labeling emotions interleaved with 8 control blocks. Each matching and labeling block consist of 8 stimuli of 3500 ms duration and control block consisted of 4 stimuli of the same duration. Total block duration of matching and labeling task is 28 seconds and that of control block is 14 seconds. Total scan duration is 7:10 min. The blocks are presented in a pseudo randomized manner. Two paradigms are developed with a change in the order of the appearance of blocks. The appearance of stimuli within blocks is randomized. The paradigm is counterbalanced across subjects with respect to the type of emotions, the intensity of emotions and the sex and the age of the images. The paradigm begins with a control block followed by either matching or labeling task. The EPI sequence parameters are: TR = 2000ms; TE = 28 ms; flip angle = 80; slice thickness = 3 mm; Slice order: ascending; slice-number = 42; Gap = 0.3 mm; Matrix = 64 × 64 × 64 mm^3^, FOV=217 × 217 × 142 mm, voxel = 3.0 mm isotropic with 208 dynamic scans.

##### 2.5.1.3 Task fMRI-2: tool for recognition of emotions in neuropsychiatric disorder (TRENDS) paradigm

The experimental paradigm consists of human faces depicting fear, anger, sad, disgust, and neutral emotions adapted from our previous publication.^[25]^ The faces are projected on a screen and subjects are instructed to passively view the images. The emotions are projected sequentially in a block design in the following order, Neutral, Sad, Disgust, Fear, and Anger. Each block consists of 8 stimuli consisting of an equal number of old and young men and women faces (high resolution 1920 × 1080 images). The first 4 stimuli are of mild intensity emotions and the next 4 stimuli are of higher intensity emotions. Each stimulus is displayed for 3500 ms duration interspersed with 500 ms of crosshair between each stimulus. The duration of each block is 32 seconds and these 5 blocks are repeated twice amounting to a total of 80 stimuli displayed over 5.3 minutes (320 seconds). The scan acquisition parameters are: TR = 2000 ms; TE = 28 ms; flip angle = 80; slice thickness = 3 mm; slice order: ascending; slice-number = 42; Gap = 0.3 mm; Matrix = 64 × 64 × 64 mm^3^, FOV = 217 × ∗217 × 142 mm, voxel = 3.0 mm isotropic with 160 dynamic scans.

##### 2.5.1.4 Diffusion tensor imaging

DTI data is acquired to analyze the white matter metrics. To correct for potential geometric distortions during image processing, reference images (*b* = 0) are acquired in opposite phase-encoding directions (posterior-to-anterior and anterior-to-posterior). To minimize signal loss in susceptible regions, shimming is applied (similar to fMRI protocol). DTI is acquired with a-shell sampling protocol as described earlier.^[36]^ This DTI protocol ensures optimal angular coverage with resultant better angular resolution. The DTI parameters are: TR = 7226 ms, TE = 100ms, FOV = 240 × 240 × 140 mm, Voxel-size = 2.5 mm × 2.5 mm × 2.5 mm, Slice-thickness = 2.5 mm, no inter-slice gap, number of slices = 56, B0 = 7, B1000 = 25, B2000 = 24, B3000 = 24. In addition to this, a low *b*-value shell 500 smm^−2^ with 6 gradient directions are also acquired.

The fMRI processing and analyses will be carried out for all subjects using Statistical Parametric Mapping (SPM) software utilizing standardized processing steps (http://www.fil.ion.ucl.ac.uk/spm/). SPM combines the General Linear Model and Gaussian field theory to draw statistical inferences from blood oxygenation level-dependent (BOLD) response data regarding deviations from the null hypothesis in 3-dimensional brain space.^[37,38]^ The images will be subjected to rigorous quality control assessments. Artefact-free, good quality data will be subjected to standard pre-processing and first-level design specification analysis. This will be followed by second-level random effect analyses to examine for differences in BOLD response among study groups, treatment effects (i.e., baseline versus follow-up comparison) as well as the correlation between the BOLD response and various other study parameters (clinical symptoms scores, and other neurobiological parameters). The specific contrast of interest will be BOLD signal differences during the performance of emotion processing tasks between the 2 groups at baseline as well as analysis for interaction effects (pre versus post). The voxel-wise analysis will produce a statistical parametric map in the stereotactic space of the Montreal Neurological Institute.^[39]^ Significance corrections for multiple comparisons will be performed using a family-wise error correction (*P* < .05). The coordinates of significant areas of activation will be transformed from MNI space into the stereotactic space of Talairach and Tournoux. Resting-state functional images will be processed using the CONN functional connectivity toolbox^[40]^ and DPABI toolbox^[41]^ for analyzing the changes in functional connectivity, regional homogeneity, amplitude of low-frequency fluctuation (ALFF), and fractional ALFF. DTI will be processed using the tract-based spatial statistics pipeline^[42]^ to examine for changes in fractional anisotropy and diffusivity measures in white matter tracts.

Table 1 gives the schedule of assessments in the study.

**Table 1:**
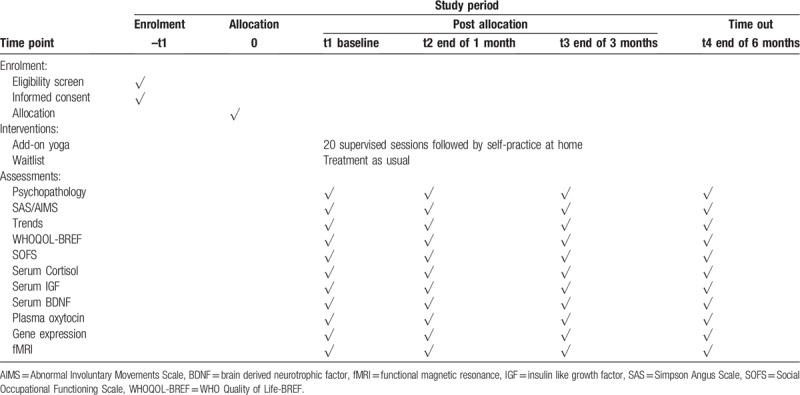
Participant enrolment and schedule of assessment.

## 3 Discussion

The findings of the proposed study are expected to have a major impact on establishing the scientific basis for the effects of yoga in a severe mental disorder such as schizophrenia. The results will have the potential to significantly impact the use of yoga in clinical management of schizophrenia and psychosis in India and the world.

## Acknowledgments

The authors wish to acknowledge the contribution of Dr Raghavendra Kumar in helping with the initiation of the study and staff from the Translational Psychiatry Laboratory at NIMHANS for helping with the imaging and laboratory work.
